# Species–habitat associations in an old-growth temperate forest in northeastern China

**DOI:** 10.1186/s12898-018-0177-9

**Published:** 2018-07-09

**Authors:** Qi Liu, Lianzhu Bi, Guohua Song, Quanbo Wang, Guangze Jin

**Affiliations:** 1 Center for Ecological Research, Northeast Forestry University, Harbin, 150040 China; 2 Heilongjiang Fenglin National Nature Reserve Authority, Yichun, 153033 China

**Keywords:** Mixed broadleaved-Korean pine forest, Topography, Niche theory, Life stages, Life forms, Shade tolerance, Habitat associations

## Abstract

**Background:**

Species coexistence mechanisms and maintenance of biodiversity have long been considered important components of community ecology research. As one of the important mechanisms, species coexistence theory based on niche differentiation has received attention in past years. Thus, topography, through the formation of habitat heterogeneity, affects species distributions and coexistence. A 30-ha dynamic plot of mixed broadleaved-Korean pine (*Pinus koraiensis*) forest is located in the Heilongjiang Fenglin National Nature Reserve. We examined species–habitat associations using the torus-translation method. We aim to understand the habitat associations of different species, life forms (shrubs, trees), and shade tolerance (light-demanding, midtolerant, shade-tolerant) across life stages (sapling, juvenile and mature), providing further evidence for the role of niche theory in temperate forests.

**Results:**

Of the 33 species we tested, 28 species (84.8%) were at least significantly associated with one habitat type. Positive associations were more frequent in the valley and slope (shady and sunny) and less frequent on the ridge. Thirty-four significant positive associations with the five habitats were detected at three life stages (11, 11 and 12 at the sapling stage, juvenile stage, and mature stage, respectively). The trees were positively associated with the valley, and the shrubs were positively associated with sunny and ridge. The majority of species’ habitat preferences shifted among different life stages; the exceptions were *Corylus mandshurica*, *Maackia amurensis*, *Quercus mongolica*, *Picea jezoensis* and *Acer ukurunduense*, which had consistent associations with the same habitat at all stages. The midtolerant trees and midtolerant shrubs were positively correlated with sunny across the three life stages.

**Conclusions:**

Most species show habitat preferences in the plot. These results indicate that niche theory plays an important role in species coexistence. Most species have no consistent association with habitat at different life stages.

## Background

Understanding the coexistence mechanisms of different species in communities is a central theme in community ecology [1, 2]. Among the many hypotheses regarding the mechanisms, the most relevant are niche theory and neutral theory [3, 4]. Niche theory holds that different species have their own niches and are limited by different ecological factors. Species coexist by occupying different resources, time and space [5]. Niche theory emphasizes the deterministic process of community assembly. Species adaptation to specific conditions determines the distribution of different species along the environmental gradient in space and time [2, 6]. Therefore, in a community dominated by niche theory, different species will evolve and adapt to their specific environment and thus show habitat associations [7, 8]. Species are competitively dominant and relatively more abundant in their suitable habitat, and abiotic factors, such as light, water and soil nutrients, can potentially influence species distributions, coexistence and diversity [9, 10]. Some studies have suggested that species composition in temperate forests may be determined by niche rather than by chance [1, 11]. Therefore, niche theory plays a major role in species coexistence in temperate forests [12].

Studies have shown that abiotic filtering should be more apparent on larger spatial scales, and biotic determinism is more important on smaller spatial scales [13]. Habitat heterogeneity, influenced by topography and other environmental factors, is considered a major factor affecting species distribution [14, 15]. Topography through geomorphic processes influences soil, water, heat, and nutrient redistribution leading to the heterogeneity of habitat resources and therefore has a spatial effect on the redistribution of vegetation to facilitate species coexistence [16]. Environmental heterogeneity promotes species diversity in three ways [17]: increasing environmental changes and resources to enable more species to coexist [18]; providing shelter for adverse conditions and protects species survival [19]; and increasing species formation with increasing heterogeneity [20]. Environmental variables are always differentiated along environmental gradients, and their roles are generally reflected in species distributions due to species–habitat associations [21]. Habitat association occurs at 1–50 ha [21, 22], and most species are likely to show habitat association in natural forests [23]. The association between species and habitats is the simplest utilized method to reflect niche theory [6]. Habitat associations of different species are related to habitat heterogeneity; therefore, we use this method to help us understand the contribution of habitat niche differentiation to the maintenance of species coexistence [24, 25].

With the development of niche theory, Grubb proposed the regeneration niche theory. The theory holds that species life strategies are different; the conditions of their seed production, spread, and germination are also different; and species are compensated by favourable regeneration conditions when their trophozoites are competing unfavourably [26]. A large amount of research has observed that the habitat preferences of species will change throughout different life stages [22, 27]. The reason for the change may be that the accumulation of environmental filters will increase with age or because of seed dispersal, density-dependence [22, 28] or other factors. Metz concluded that species had greater niche differentiation at later life stages due to different habitat mortality rates [29]. Therefore, dispersal limitation played an important role in the distribution of small trees, and environmental change played an important role in the distribution of large trees [30]. Understanding the association of different life stages helps us develop a more comprehensive tree species coexistence mechanism.

Most studies focusing on habitat associations were concentrated in tropical forests, and few studies have been performed in temperate forests [21, 24, 25]. This study was conducted in an old-growth temperate forest, and the mixed broadleaved-Korean pine forest is the zonal climax vegetation in the eastern mountainous area of northeast China, and is representative of a temperate conifer and broad-leaved mixed forest. This forest is well known for its unique species, species diversity, and subtropical composition. In this study, 33 species of woody plants with diameter at breast height (DBH) ≥ 1 cm and more than 30 individuals were selected as the study objects in the 30-ha dynamics plot of mixed broadleaved-Korean pine forest in the Fenglin plot. The torus-translation test was used to analyse the association of different species, life stages (sapling, juvenile and mature), life forms (shrubs, trees) and shade tolerance (light-demanding, midtolerant, shade-tolerant) in five habitats. The aims of this study were (1) to investigate the habitat association of major species; (2) to examine the change of habitat associations across different life stages; and (3) to discuss the effect of niche theory in contributing to species coexistence in temperate forests. This study will provide an important scientific basis for the mechanism that promotes species coexistence in temperate forests.

## Methods

### Study site

The study was carried out in an old-growth temperate forest, which was a 30-ha Forest Dynamics Plot in the Heilongjiang Fenglin National Nature Reserve, Yichun City, Heilongjiang Province, Northeast China. The total area is 18,165 ha. The site is a temperate conifer and broad-leaved mixed forest, the mean annual temperature is − 0.5 °C, and it is affected by the East Asian ocean currents and the Siberian cold current double impact with continental and monsoon climate characteristics and long winters but warm, transitory summers. The mean annual precipitation is 688 mm, mostly falling from June to September. The relative humidity is 73%, frost-free days per year are 120, and growth days per year are 100. The dominant soil is dark brown soil, and the soil depth is 20–50 cm. The region belongs to lowland hilly terrain and is surrounded by water. The main rivers are the Tangwang River, Fenglin River, Pingyuan River and nine other rivers. The seasonal flowing water running through the region constitutes a complete water system. The forest coverage is 95%, which is one of the oldest virgin forests in the broadleaved-Korean pine forest ecosystem. The plot has a complex community composition and high species diversity, which is dominated by *Pinus koraiensis* and coupled with the broad-leaved species of *Acer tegmentosum*, *Fraxinus mandshurica*, *Acer mono*, *Tilia amurensis*, and *Ulmus laciniata*, as well as the conifer species *Abies nephrolepis*, *Picea koraiensis*, and *Picea jezoensis*.

### Data collection

**Fig. 1:**
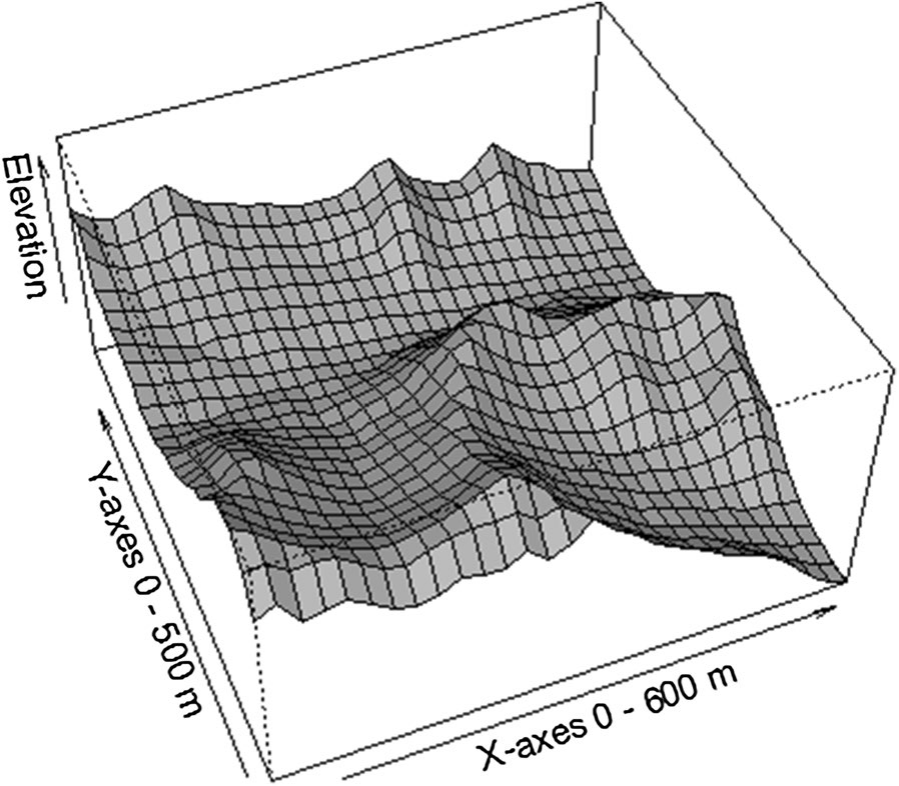
Topographic map with 20-m intervals of the 30-ha Fenglin permanent plot in Xiaoxinganling, Northeast China

In 2009, a 30-ha (600 m × 500 m) permanent plot was established (Fig. 1). The total station was used to divide the plot into 750 20 m × 20 m quadrats. All woody stems with DBH ≥ 1 cm in the plot were identified, measured, had their status recorded (survival, lodging, standing die), mapped and tagged. A recensus was conducted in 2014.

In 2014, approximately 75,586 individuals had a DBH ≥ 1 cm in 22 families, with 37 genera and 44 species. Approximately 79% of the measured woody plants had a DBH of less than 5 cm, whereas 2.73% had a DBH of more than 40 cm. The most abundant species was *Corylus mandshurica*, accounting for 25.3% of the total individuals. This abundance was followed by *Actinidia kolomikta*, *Abies nephrolepis*, *Acer ukurunduense*, *Acer tegmentosum*, *Acer mono*, *Lonicera chrysantha*, *Pinus koraiensis*, *Acanthopanax senticosus*, and *Betula costata*. The total number of these 10 species accounted for 82.98% of the total number of individuals in the plot. There were 8 species with fewer than 10 individuals, accounting for 18% of the total species.

### Habitat classification

**Fig. 2:**
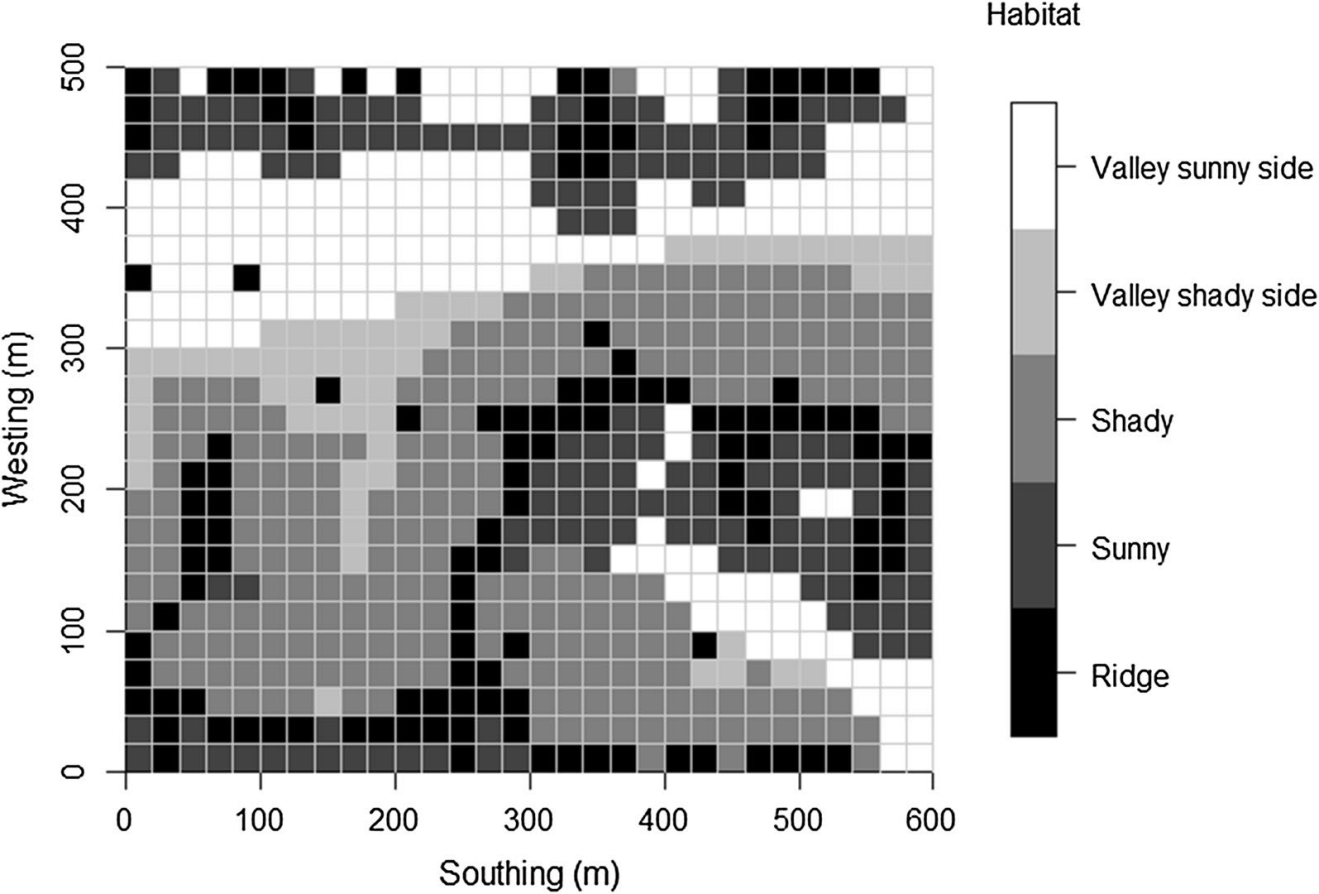
The 30-ha Fenglin permanent plot, divided into habitats assigned to 20 m × 20 m quadrats

The altitude of the four corners of each quadrat were made into a digital elevation model (DEM), and the elevation, slope, aspect, and curvature were derived from the DEM. Aspect was a circular variable, so aspect was transformed to sin (aspect) and cos (aspect) [27]. We used a multiple regression tree (MRT) [31] to divide the 750 quadrats into five habitat types containing similar topographical classifications and species compositions [32]. The multiple regression tree is a statistical technique that can predict the relationship between species and environmental factors [31]. Using the recursive partitioning analysis, the quadrats were divided into homogeneous categories to the greatest extent possible. Tree size was selected by minimizing the cross-validated relative error (CVRE) with 1 SE rules; thus, it was more objective than the common method. Four topographic attributes of 20 m × 20 m quadrats were selected as independent variables. The importance value (IV) of 44 species in 750 quadrats was the dependent variable, and the importance value was calculated as IV = (relative abundance + relative basal area)/2. Categorization was accomplished by the “mvpart” library in R [32]. The 750 quadrats were assigned to five topographic habitat types: ridge, sunny, shady, valley shady side, valley sunny side (Fig. 2). The specific parameters of the five habitat types are shown in Table 1.

**Table 1:** Topographic parameters of the five habitat categories in the 30-ha Fenglin permanent plot

Habitat types	Number of quadrats	Total area (ha)	Relative elevation (m)			Slope (°)			Aspect (°)			Curvature (m)		
			Min.	Max.	Mean	Min.	Max.	Mean	Min.	Max.	Mean	Min.	Max.	Mean
Ridge	125	5	416.5	489.6	452.9	2.4	25.9	12.9	2.7	359.8	158.9	0.9	5.5	2.3
Sunny	145	5.8	405.8	480.7	437.6	9.2	22.9	14.0	91.8	258.8	161.9	− 5.5	0.7	− 0.9
Shady	247	9.88	407.6	480.7	442.0	9.2	25.3	14.9	0.5	357.6	130.1	− 2.4	0.8	− 0.3
Valley shady side	61	2.44	408.6	463.9	428.1	1.6	9.0	6.0	0.0	359.5	85.3	− 2.2	0.7	− 0.6
Valley sunny side	172	6.88	401.4	453.3	427.7	1.4	9.2	6.5	86.9	276.4	141.5	− 2.3	0.8	− 0.2

### Analyses of species–habitat associations

Only those species represented by more than 30 individuals within the plot were included in the analysis. The torus-translation test has proved to be useful in comparing species–habitat associations [21]. The concept is to calculate the probability of a species in a habitat under a random distribution and to determine whether a species is significantly related to this habitat by chance. In contrast to conventional methods, this method is able to consider the spatial autocorrelation of the species distribution, making the test more sensitive [21]. The true habitat map was moved in four directions (up, down and left, right) by 20-m increments, 30 times in the horizontal direction and 25 times in the vertical direction. The horizontal and vertical directions can be moved at the same time. A total of 30 × 25 relative densities were obtained. Three maps are generated by a 180° rotation, mirror image and a 180° rotation of the mirror image, so each species can have 3000 relative densities (2999 simulated relative densities and 1 true relative density). Comparing the position of the true relative density in the simulated relative densities, if it is > 97.5%, then the species is statistically positively associated with this habitat. If it is < 2.5%, then the species is statistically negatively associated with this habitat. If it falls in the middle, then there is no correlation (α = 0.05 significant level, two-tailed test).

Under the same environmental conditions, the diameter and life stages of the same species have the same response to the environment [33], so we used the DBH to represent different life stages [34]. According to DBH, we divided species into three life stages (sapling, juvenile and mature) (Table 2). We analysed species with more than 30 individuals in each of the three life stages. Nineteen species conform to the requirements. To compare the habitat associations at the three life stages more accurately, the absolute stem density of the species is used instead of the relative density of the species (α = 0.01 significant level, single-tailed test). We only considered positive associations [22].

**Table 2:** Life stage classifications based on DBH for species of different life forms: shrubs and trees

	Life stages	DBH (cm)
Trees	Sapling	1.0 ≤ DBH < 5.0
	Juvenile	5.0 ≤ DBH < 10.0
	Mature	DBH ≥ 10.0
Shrubs	Sapling	1.0 ≤ DBH < 2.0
	Juvenile	2.0 ≤ DBH < 3.0
	Mature	DBH ≥ 3.0

To understand the preference of different species for habitat, the species were divided into different life forms (shrubs and trees), shade tolerance (light-demanding, midtolerant, and shade-tolerant) [35], and life stages.

## Results

### Associations of different species, life forms, and shade tolerance with five habitat types

According to the torus-translation test (*P* < 0.05), 28 of the 33 species exhibited significant positive or negative associations with at least one habitat type. *Picea koraiensis*, *Phellodendron amurense*, *Aralia elata*, *Viburnum burejaeticum*, and *Populus davidiana* did not show significant associations with any type of habitat.

In positive associations, the number of species related to sunny and valley shady side habitat was the highest (9 species), and the ridge was least (5 species). In the negative associations, more species were found in the sunny (13 species), and less species were found in the valley shady side (6 species). From the distribution pattern, the size of the habitat area had no effect on the number of habitat associations. Although a species is positively associated with one habitat, it is not completely negatively associated with the other four habitats and is not completely excluded from the other four habitat types (Table 3).

**Table 3:** Species habitat-associations in the 30-ha Fenglin permanent plot

Species	Ridge	Sunny	Shady	Valley shady side	Valley sunny side
*Syringa reticulata* var*. mandshurica*	N	−	N	+	−
*Sambucus williamsii*	−	−	N	+	N
*Prunus padus*	−	N	N	N	+
*Abies nephrolepis*	−	N	N	+	N
*Acanthopanax senticosus*	N	−	+	N	N
*Ribes mandschuricum*	−	−	+	N	+
*Philadelphus schrenkii*	N	−	+	+	N
*Betula costata*	−	N	N	+	+
*Actinidia kolomikta*	−	−	+	N	N
*Deutzia glabrata*	−	N	+	−	N
*Picea koraiensis*	N	N	N	N	N
*Pinus koraiensis*	+	+	−	N	N
*Acer ukurunduense*	−	−	N	N	+
*Sorbus pohuashanensis*	N	+	N	−	N
*Phellodendron amurense*	N	N	N	N	N
*Lonicera chrysantha*	N	−	+	+	N
*Ulmus laciniata*	+	−	N	N	−
*Euonymus pauciflorus*	N	+	N	N	N
*Aralia elata*	N	N	N	N	N
*Alnus sibirica*	N	−	−	+	+
*Corylus mandshurica*	+	+	N	−	−
*Quercus mongolica*	N	+	−	−	−
*Viburnum burejaeticum*	N	N	N	N	N
*Acer tegmentosum*	−	N	N	N	+
*Acer mono*	+	+	−	N	−
*Rosa acicularis*	−	N	+	N	N
*Maackia amurensis*	N	+	−	−	−
*Populus davidiana*	N	N	N	N	N
*Fraxinus mandshurica*	N	−	N	+	N
*Picea jezoensis*	−	−	N	+	+
*Sorbaria sorbifolia*	−	−	N	N	+
*Tilia amurensis*	+	+	−	N	−
*Rhododendron dauricum*	N	+	−	−	N
Positive association	5	9	7	9	8
Negative association	12	13	7	6	7
Total	17	22	14	15	15

+, −, and N indicate positive associations, negative associations, and no corresponding associations, respectively

Shade-tolerant species were positively associated with shady and valley and negatively associated with ridge and sunny. Light-demanding species were positively associated with ridge and valley shady side and negatively associated with shady. Midtolerant species were positively associated with ridge and sunny and negatively associated other habitats. Trees were positively associated with valley, and shrubs were positively associated with ridge and sunny (Table 4).

**Table 4:** Species–habitat associations with different life forms and shade tolerance in the 30-ha Fenglin permanent plot

	Ridge	Sunny	Shady	Valley shady side	Valley sunny side
Shade-tolerant shrubs	N	−	+	N	N
Light-demanding shrubs	−	N	N	N	+
Midtolerant shrubs	+	+	N	−	−
Shade-tolerant trees	−	N	N	+	+
Light-demanding trees	+	N	−	+	−
Midtolerant trees	N	+	−	N	+
Shade-tolerant	−	−	+	+	+
Light-demanding	+	N	−	+	N
Midtolerant	+	+	−	−	−
Trees	N	N	−	+	+
Shrubs	+	+	N	−	−

+, −, N indicate positive associations, negative associations, and no corresponding associations, respectively

### Association of different species, life forms, and shade tolerance in different life stages with five habitat types

Based on the torus-translation test (*P* < 0.01), there were 34 significant positive associations among the 5 habitats and 16 species at three life stages. Among the 34 positive associations, 11 were detected at the sapling and juvenile stages, and 12 occurred at the mature stage. *Picea koraiensis* and *Fraxinus mandshurica* did not exhibit habitat associations at any of the three life stages.

**Fig. 3:**
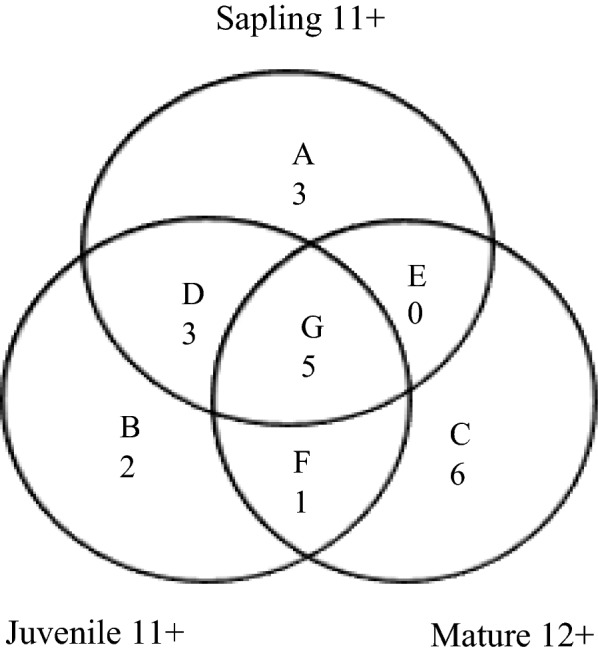
Venn diagrams illustrating positive associations of the sapling, juvenile, and mature stages in the five habitats in the 30-ha Fenglin permanent plot. Significance of association was determined with torus-translation tests at P < 0.01. The number of positive associations is the number of species. A, B, and C represent only one positive association at any life stage. D, E, and F are positive association at both stages. G is simultaneous positive associations at all three life stages

The results showed that *Corylus mandshurica*, *Quercus mongolica*, *Populus davidiana*, *Picea jezoensis* and *Acer ukurunduense* were associated with the same habitat at three life stages (G pattern in Fig. 3). Three positive associations appeared at both sapling and juvenile stages but changed at the mature stage (D pattern in Fig. 3). One species maintained the same habitat association during the juvenile and mature stages but had no corresponding associations at the sapling stage (F pattern in Fig. 3). No species had the same positive association at the sapling and mature stages (E pattern in Fig. 3). Three, two, and six species showed positive associations only at sapling, juvenile, and mature stages (A, B, and C patterns in Fig. 3), respectively. *Ulmus laciniata* was positively associated with ridge at the juvenile stage and positively associated with shady at the mature stage.

**Fig. 4:**
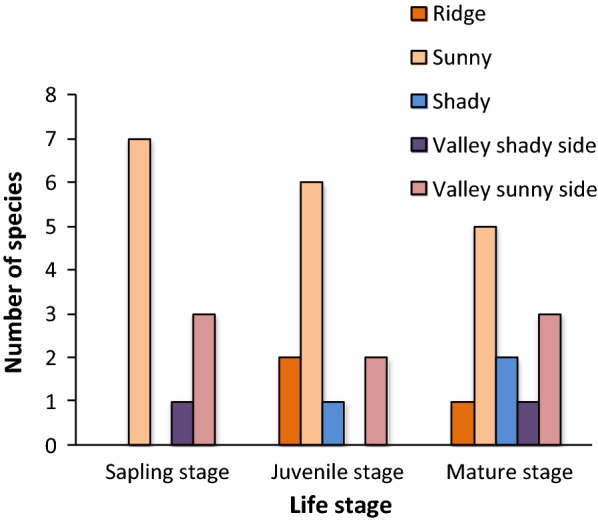
Number of species associated with each of the five habitat types at the sapling, juvenile, and mature stages in the 30-ha Fenglin permanent plot

The number of species that were positively associated with the five habitats was different across life stages. At the three life stages, most of the species were associated with sunny. At the sapling stage, no species were observed to be associated with ridge and shady. At the juvenile stage, no species were found to be associated with the valley shady side (Fig. 4).

The association of midtolerant shrubs and midtolerant trees with sunny was consistent across the three life stages. Shrubs had a positive association with sunny at all three life stages, while trees were associated with sunny at the sapling and mature stages (Table 5).

**Table 5:** Habitat associations of different shade tolerance and life forms in three life stages within the 30-ha Fenglin permanent plot

	Ridge	Sunny	Shady	Valley shady side	Valley sunny side
Sapling of shade-tolerant shrubs	\	\	+	\	\
Juvenile of shade-tolerant shrubs	\	\	+	\	\
Mature of shade-tolerant shrubs	\	\	\	\	\
Sapling of light-demanding shrubs	\	+	\	\	\
Juvenile of light-demanding shrubs	\	\	\	\	\
Mature of light-demanding shrubs	\	\	\	\	\
Sapling of midtolerant shrubs	\	+	\	\	\
Juvenile of midtolerant shrubs	\	+	\	\	\
Mature of midtolerant shrubs	\	+	\	\	\
Sapling of shade-tolerant trees	\	\	\	\	\
Juvenile of shade-tolerant trees	\	\	\	\	\
Mature of shade-tolerant trees	\	+	\	\	\
Sapling of light-demanding trees	\	\	\	\	\
Juvenile of light-demanding trees	+	\	\	\	\
Mature of light-demanding trees	+	\	\	\	\
Sapling of midtolerant trees	\	+	\	\	\
Juvenile of midtolerant trees	\	+	\	\	+
Mature of midtolerant trees	\	+	\	\	\
Sapling of shrubs	\	+	\	\	\
Juvenile of shrubs	\	+	\	\	\
Mature of shrubs	\	+	\	\	\
Sapling of trees	\	+	\	\	\
Juvenile of trees	\	\	\	\	\
Mature of trees	\	+	\	\	\

+ indicates positive associations

## Discussion

### Associations of different species, life forms, shade tolerance with five habitat types

The ability of species to adapt to environmental conditions is different, which may lead to different distribution patterns of species in the environment [36]. Species that are positively associated with a habitat are more competitive than negative and no correlation species [37]. Shade tolerance is also considered to have an impact on the distribution of species [36]. *Pinus koraiensis* and *Tilia amurensis* often grow in well-drained, moist areas [38]. *Quercus mongolica* is a light-demanding species and occurs more in sufficient sunlight areas. *Abies nephrolepis* and *Picea jezoensis* are shade-tolerant species and occur more in the valley [35]. The shade-tolerant species were positively associated with less well-lit habitats. The population of shade-tolerant species can maintain or even increase under natural conditions [37]. Light-demanding species and midtolerant species were positively associated with better-lit habitats. The distributions of species were determined by the characteristics of the species. Species characteristics have their own different niches by occupying different space to form coexistence. Shrubs had a preference for well-drained soil and a strong drought tolerance [16]. Sunny and ridge habitats have enough light and are well-drained; thus, this habitat was more suitable for shrubs, while the distribution of shrubs will also be affected and restrained by trees [39]. Canopy species were mostly affected by the topography, while understory species were mostly affected by the forest structure [40]. Trees were mostly distributed in the valley, where shrubs were shaded by trees, affecting the regeneration and growth of shrubs. The functional traits of species, such as the canopy layer and shade tolerance, were important factors affecting species distributions in the CBS temperate plot [36].

In mature boreal forests, interactions between species above 10 m are independent, and the distribution patterns of trees are affected by environmental factors when this scale is exceeded [41]. The spatial heterogeneity at a large scale may be caused by environmental factors such as geology, topography and historical events [6]. Topography is a good predictor of habitat for plants, especially trees [42]. Topography via geomorphic process influences soil, water, heat and nutrient redistribution to form heterogeneous habitat resources [16]. Many studies have verified topographic niche partitioning [21, 22, 24]. In tropical forests, the distribution of species varies greatly as a result of drought sensitivity [43]. In the Korup plot, there was a sharp contrast to the number of habitat associations with valley and ridge habitats, suggesting that niche differentiation of edaphic variables contributed to species distributions and maintained species diversity in African forests [25]. In this study, most of the species were identified in the valley, and fewer species were positively associated with the ridge, which may be because the ridge is characterized by less water, phosphorus, nitrogen and other resources than the valley [44]. Relatively more species preferred the slope, which may be because the steeper areas also have sufficient water, lower nutrient loss for species growth and generally have more soil water and available nutrients than ridge areas.

Habitat has a strong effect on tree species distribution; when the habitat is complex, the proportion of habitat associations is higher. The results revealed that there were 28 positive species, accounting for 84.8% of the tested species, and 27 negative species, accounting for 81.8%. Most species exhibited habitat associations. In a 50-ha BCI plot with little topographic change, 64% of species were positively or negatively associated with at least one habitat, and habitat specialization had a limiting effect on maintaining the species diversity [21]. In a 50-ha Korup plot, 63% of species were associated with at least one habitat type, and the results showed that niche differentiation with respect to edaphic variables contributed to the maintenance of diversity in African forests [25]. In slightly more complex topography in the Jiaohe 21.12-ha plot, 72.3% species were significantly associated with at least one habitat, the results indicated that habitat differentiation contributed to the maintenance of the distribution of species [37]. In a topographically complicated Malaysian 52-ha Lambir plot, of the 10 Sterculiaceae species, 8 species exhibited significant associations with at least one habitat type; these results suggested that edaphic niche differentiation contributed to the coexistence of Sterculiaceae [45]. In a 15-ha maximum height difference Nonggang plot, 85.1% of species examined were associated with at least one habitat; thus, in the karst seasonal rain forest, niche differentiation caused by topography played an important role in the maintenance of species diversity due to the uniqueness of the ecosystem [24]. Since environmental variables are always spatially aggregated, their effects will be reflected in species distributions through species–habitat associations [21, 46]. The proportion of habitat associations are different, which may be due to the changes in environmental factors [6]. Topographic niche differentiation is enhanced with an increase in environmental heterogeneity [47]. The Fenglin plot had a higher percentage of habitat associations, with many species exhibiting habitat preferences. Environmental variables of spatial structures are related to niche processes [48], so we posit that niche differentiation plays an important role in the maintenance of species diversity. In this study, we only considered the effects of topographic factors on species distribution. We need to take into account more factors, such as soil and hydrologic factors, in future studies.

### Habitat associations change at different life stages

Species may experience a wide range of environments in their lives, which means that the regeneration stages may be different from the later or more mature stages of the environment [49]. Most species showed different habitat associations at the three life stages, indicating that habitat associations can contribute to regeneration niche and affect species coexistence [50]. For the 19 species, only 5 species showed the same habitat associations at all three life stages. At the sapling stage, 72.7% of species associations were maintained to the juvenile stage, and only 45.5% were consistent at the mature stage. These results demonstrated that habitat preferences of most species in the plot changed with different life stages. These results were largely consistent with previous studies. In a Bornean forest, only 9.1% of species maintained the same habitat preferences at multiple life stages [28]. In the BCI plot, only 6.3% species showed the same habitat preferences at different life stages [22]. In the Gutianshan plot, 26.7% of habitat associations did not change during the three life stages, indicating that the environment of the early stage for most species was not always suitable for the subsequent stage [27].

*Corylus mandshurica*, *Quercus mongolica* and *Picea jezoensis* were positively associated with the same habitat at the three life stages. These 3 species spread through gravitation, and seed dispersal led to spatial limitations and maintained their limited distributions within the same habitat, which was close to the parent tree [28], thereby facilitating the maintenance of the same association at all three life stages. Midtolerant trees and midtolerant shrubs maintained a positive association with sunny at the three life stages. The midtolerant species’ demands for light are between shade-tolerant and light-demanding species; therefore, shade-tolerant and light-demanding species will be more susceptible to the effects of habitat factors than midtolerant species due to these species characteristics. The seed dispersal ability of understory species is weaker than that of overstory species [36]. Therefore, because the seed dispersal ability of shrubs is poor, they maintained the same association at different life stages.

Habitat heterogeneity, dispersal limitation, and biotic interactions can be used to explain species distributions [14, 21]. Relevant research has shown that seed dispersal limitation may also result in changes in habitat associations at different life stages [22, 28]. Dispersal limitation can cover the effect of habitat preference on species distribution to some extent [49]. Differences in the relative importance of environmental factors and dispersal limitation are usually caused by differences in spatial scales or environmental complexity [32, 46]. In early tree stages, the effect of dispersal on species composition is expected to be higher because seed dispersal leads the recruits to a new place before environmental effects begin to act [49]. The environmental effect is small, and the nutrient requirements of saplings can be supplied by themselves in the early stage [51]. As individuals die in unfavourable habitats, the role of environmental effects in tree species distributions is more pronounced [52]. Studies have shown that the distribution of adult tree species may be explained in part by differences in growth and mortality at the seedling and sapling stages in different habitats [53]. The effect of dispersal limitation decreases with the life stage, and the environmental effect increases with the life stage [54]. Due to the dispersal limitation, saplings are mostly distributed around the parent tree. With the growth of trees, the impact of the topography on species distribution is intensified, and the selection of suitable habitat leads to the change of association [30]. It is also possible that the association may change due to negative density dependence, which will reduce the habitat association with later growth [27]. Temperate and tropical studies have shown that seedling survival and seed-to-seedling transition rates were usually due to density and distance dependence, leading to changes in species distributions from one life stage to later stages [55, 56]. The habitat preferences of species will change at different life stages, which may be due to habitat heterogeneity, seed dispersal, negative density dependence or other reasons. Therefore, it is necessary to perform further investigations of seedlings with DBH < 1 cm.

## Conclusions

In this paper, we studied the mechanism of topographic factors in shaping species distribution patterns in a mixed broadleaved-Korean pine forest. The majority of species showed habitat associations, indicating that niche partitioning caused by topographical heterogeneity played an important role in shaping species distributions and coexistence in the Fenglin plot. Our analyses suggest that the habitat preference of most species changed at different life stages. Species with the same niche requirements can contribute to coexistence by altering habitat preferences.
